# Plant grafting: insights into tissue regeneration

**DOI:** 10.1002/reg2.71

**Published:** 2016-12-21

**Authors:** Charles W. Melnyk

**Affiliations:** ^1^ The Sainsbury LaboratoryUniversity of CambridgeBateman StreetCambridgeCB2 1LRUnited Kingdom

**Keywords:** *Arabidopsis*, callus, grafting, horticulture, regeneration, vasculature, wounding

## Abstract

For millennia, people have cut and joined different plants together through a process known as grafting. The severed tissues adhere, the cells divide and the vasculature differentiates through a remarkable process of regeneration between two genetically distinct organisms as they become one. Grafting is becoming increasingly important in horticulture where it provides an efficient means for asexual propagation. Grafting also combines desirable roots and shoots to generate chimeras that are more vigorous, more pathogen resistant and more abiotic stress resistant. Thus, it presents an elegant and efficient way to improve plant productivity in vegetables and trees using traditional techniques. Despite this horticultural importance, we are only beginning to understand how plants regenerate tissues at the graft junction. By understanding grafting better, we can shed light on fundamental regeneration pathways and the basis for self/non‐self recognition. We can also better understand why many plants efficiently graft whereas others cannot, with the goal of improving grafting so as to broaden the range of grafted plants to create even more desirable chimeras. Here, I review the latest findings describing how plants graft and provide insight into future directions in this emerging field.

## 1 INTRODUCTION

**Figure 1:**
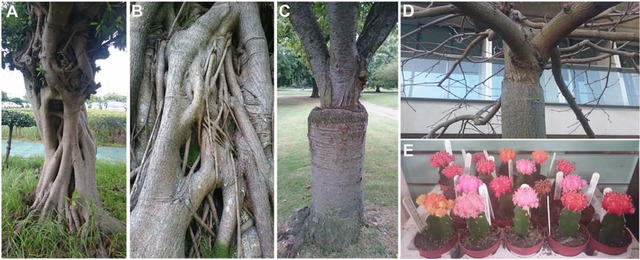
**Grafting in nature and in horticulture**. (A), (B) *Ficus virens* trees whose branches and stems naturally self‐graft through contact and fusion of tissues. (C) Two varieties of *Prunus* grafted together. (D) Two varieties of *Tilia* grafted together. (E) Grafts between a colorful cactus on top (*Gymnocalycium*) and a green cactus in the stalk (species vary).

Plants have an incredible capacity for regeneration since, as sessile organisms, they need to repair damage from a variety of biotic and abiotic stresses. They can effectively heal cuts and wounds, completely regenerate an excised root tip, and form new organs and tissues when placed on growth media containing high levels of plant hormones (Birnbaum & Sanchez Alvarado, 2008; Sugimoto, Gordon, & Meyerowitz, 2011). One striking example of this phenomenon occurs during grafting when two plants are cut and joined together. Tissues and vasculature regenerate around the cut site as the shoot (called the scion) and stock form a chimeric individual (Box 1, Fig. 1) (Melnyk & Meyerowitz, 2015). This fascinating process is an unusual example in biology of two different individuals becoming a single chimera. The process is not always artificial, though, as in nature certain plants can fuse their tissues to each other and other plants when stems or roots come into contact (Fig. 1) (Warschefsky et al., 2016).

Despite this relevance, we still have little mechanistic idea of how plants graft. Work done since the late 1800s described a series of events that led to successful graft formation (Box 2). After cutting, tissues adhere and cell expansion and division leads to the formation at the wound site of undifferentiated stem‐cell‐like tissue called callus (Birnbaum & Sanchez Alvarado, 2008; Ikeuchi, Sugimoto, & Iwase, 2013). Callus and tissues surrounding the cut differentiate to phloem and xylem before the vascular strands are connected between scion and stock (Box 1). Much of this work was performed on non‐model species, limiting the mechanistic insight on graft formation. Recently, though, new techniques have allowed grafting of hypocotyls, meristems, cotyledons, and inflorescences of the model plant *Arabidopsis thaliana* (Rhee & Somerville, 1995; Turnbull, Booker, & Leyser, 2002; Chen, Komives, & Schroeder, 2006; Flaishman, Loginovsky, Golobowich, & Lev‐Yadun, 2008; Nisar, Verma, Pogson, & Cazzonelli, 2012; Yoo, Hong, Jung, & Ahn, 2013; Huang & Yu, 2015), which has provided important insights into our understanding of grafting and regeneration.

Box 1. **Glossary.**

**Scion** – The upper part of the graft, typically a shoot.

**Stock** – The lower part of the graft. Typically a stem or a root, in which case it is called a rootstock.

**Phloem** – Vascular tissue that transports hormones, sugars, nucleic acids, and proteins throughout the plant. Composed of living conducting cells that lack nuclei (sieve elements) and living companion cells that support the sieve elements.

**Xylem** – Vascular tissue that provides mechanical support and transports water and nutrients throughout the plant. Composed of dead fibers that provide mechanical support, dead tracheary elements that transport water, and living parenchyma cells.

**Cambium** – Undifferentiated stem‐cell‐like tissue that divides and differentiates to give rise to xylem and phloem cells. During primary development (shoot and root tip growth), it is known as procambium. During secondary development (stem and root thickening), it is known as cambium.

**Figure 2:**
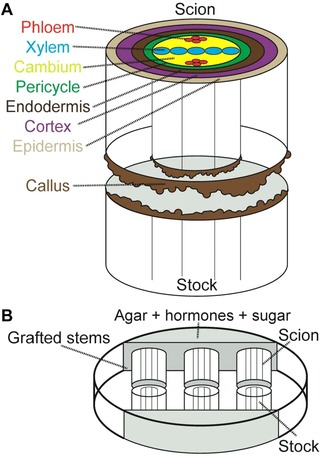
**Stem anatomy and grafting tools**. (A) The arrangement of cell types in the *Arabidopsis* root and hypocotyl; the latter tissue is commonly grafted in science. Tissues including the cortex, cambium, endodermis, and pericycle, are thought to divide and differentiate in various plant species upon cutting to heal the graft junction (Jeffree & Yeoman, 1983; Melnyk et al., 2015). Wound‐induced callus is found throughout the cut surface. Adapted from Melnyk, 2016 with permission from Wiley. (B) *In vitro* grafting assays place short segments of stock and scion together to observe the nutrient and hormone requirements for successful connection (Parkinson & Yeoman, 1982). Nutrients and hormones are placed in agar at either end of the plate. The *in vitro* grafting system is a useful tool for identifying and characterizing factors that act systemically to promote or inhibit graft formation.

**Xylem pole pericycle cells** – Pericycle cells located adjacent to the xylem (see Fig. 2). Xylem pole pericycle cells divide and differentiate to give rise to lateral roots and callus in many plant species. They may also play an important role in graft formation.

Here, I describe recent developments towards understanding how plants graft. Common themes beginning to emerge include cell division, cell wall modifications, hormone responses, and vascular differentiation. I also discuss work done on related phenomena, including vascular formation and wound healing, since fundamental processes such as cell differentiation and cell division during these processes are likely to be similar and highly informative for understanding how plants regenerate the graft junction.

## 2 THE HORTICULTURAL AND SCIENTIFIC IMPORTANCE OF GRAFTING

Grafting is extremely important in horticulture. It has been practised for millennia and likely contributed to the domestication of certain woody plants such as apples, pears, and plums by allowing the asexual propagation of desirable plants that were highly heterozygous and did not root easily from cuttings (Mudge, Janick, Scofield, & Goldschmidt, 2009). In the last century, grafting has expanded beyond woody plants so a wide variety of plants are grafted. Over 1 billion vegetable plants in Japan and Korea are grafted, primarily from the Solanaceae (i.e., tomatoes, eggplants, peppers) and melon families (Lee et al., 2010). Over 70 woody perennial crops grown for their fruits are grafted, and 20 of the 25 most produced fruit and nut crops are sometimes grafted (Warschefsky et al., 2016). Some are grafted for asexual propagation but many are grafted instead to introduce resistance to biotic stresses such as insects or to abiotic stresses such as salinity, cold, or unfavorable soil conditions (Lee et al., 2010; Garner & Bradley, 2013). A notable pathogen involved the arrival in Europe from North America of the insect phylloxera that fed on and killed European grape vines (Mudge et al., 2009). By the late 1800s, the majority of the French wine industry was decimated. The solution was to graft a phylloxera‐resistant North American grape rootstock to the European grape scion, providing effective resistance and saving the European wine industry. Today, the vast majority of wine grapes are grafted to provide phylloxera resistance (Mudge et al., 2009).

Box 2. **A time course for graft formation.**

A representative time course based on tomato and *Sedum* grafting (Lindsay, Yeoman, & Brown, 1974; Moore & Walker, 1981; Jeffree & Yeoman, 1983; Moore, 1983). The plant species and age of the tissue will affect the time taken to graft. For instance, in passion fruit the timing is slower (Ribeiro, Nery, Vieira, & Mercandante‐Simoes, 2015) whereas in young *Arabidopsis* it is faster (Melnyk et al., 2015).

Within the first day or two after grafting at the graft junction:

(1) adhesion of scion and stock; cell wall materials including pectins are secreted
(2) a necrotic layer of dead cells forms
(3) cell divisions initiate in the cambium, endodermis, cortex, and cells surrounding the phloem and xylem in both scion and stock; callus begins to form
(4) a very slow increase in breaking weight strength occurs (the amount of weight required to break apart the graft)

Several days to a week after grafting:

(5) the necrotic layer begins to fragment
(6) callus makes contact between scion and stock
(7) a common cell wall forms between the scion and stock from newly deposited cell wall material including pectins
(8) the common cell wall thins and plasmodesmata form across it between scion and stock
(9) a substantial (typically linear) increase in breaking weight strength occurs

A week or more after grafting:

(10) the callus continues to proliferate and differentiates to cambium; later, xylem and phloem elements differentiate
(11) cells surrounding the cut also differentiate to vascular tissue
(12) vascular connections are re‐established
(13) the necrotic layer is no longer visible
(14) the breaking weight strength levels off to provide strength at the graft junction similar to ungrafted material

A third important use of grafting is to change the growth habits of the scion by altering its size, controlling growth vigor, or increasing fruit yields (Mudge et al., 2009; Lee et al., 2010). For example, grafting with an M27 apple rootstock causes an apple scion to dwarf and produce a tree 30% of full size that is suitable for small gardens. Using an MM111 apple rootstock causes the same scion to produce a tree 90% of full size that is more suitable for orchards (Mudge et al., 2009). Modifying the size and the vigor of the plant is advantageous to increase yield and improve planting densities. Thus, the rootstock exerts important control over the scion and selecting the correct rootstock influences the size, the vigor, and the stress resistance of the tree. The mechanism causing these graft‐induced effects remains somewhat mysterious and probably involves differences in hormone, water, or signaling molecule production and transport in the grafted rootstock compared to the ungrafted individual (discussed in Aloni, Cohen, Karni, Aktas, & Edelstein, 2010; Warschefsky et al., 2016). Much effort is put into breeding desirable rootstocks and finding rootstocks that can successfully graft with a scion, a term referred to as compatibility (Garner & Bradley, 2013; Warschefsky et al., 2016). The reason some plants are unable to graft, such as most monocots, whereas others can easily graft has remained a mystery and older texts often give conflicting information about which plants can be grafted to which (Mudge et al., 2009).

More recently, breeding efforts and automation techniques with robots have improved grafting success rates and increased the number of grafted plants and species (Mudge et al., 2009; Lee et al., 2010; Garner & Bradley, 2013). Researchers are also investigating the basis for compatibility and incompatibility (reviewed by Pina & Errea, 2005; Goldschmidt, 2014). In addition to this, researchers have used grafting to study the long distance movement of molecules. By grafting different genotypes to each other and looking for the movement of molecules from one genotype to another, researchers have identified the transport of proteins, hormones, RNAs, and secondary metabolites over long distances (discussed in Turnbull, 2010; Goldschmidt, 2014). Grafting has also been important for identifying fundamental principles of plant development. The biologist Tsvi Sachs formulated the canalization hypotheses of the plant hormone auxin by, in part, observing the differentiation of vascular strands during plant cutting and grafting (Sachs, 1968). He proposed that auxin converged in channels that transported auxin from the shoots to the roots and that these channels would differentiate to vascular tissue (Sachs, 1981). Thus, grafting has proven incredibly useful for both horticultural and scientific applications.

## 3 INITIATING GRAFT FORMATION

After grafting, plant responses are rapidly activated as the plant detects wounding and begins the regeneration process. There are several possibilities for how a plant could sense and initiate graft formation. First, cutting the vasculature produces an asymmetry at the cut by changing transport dynamics. Various substances are mobile through plant tissues such as auxin that is transported basipetally from growing leaves to the roots (reviewed by Friml & Palme, 2002) and sugars that are transported from photosynthetic sources such as leaves to sinks such as the roots (reviewed by Stitt, 1996). Thus, severing the vascular tissues will presumably cause the accumulation of auxin and sugars above the cut site while depleting these substances below until cellular and vascular connections are restored. In cut *Arabidopsis* inflorescence stems, auxin accumulation above the cut activates *ANAC071* expression, whereas auxin depletion below the cut activates *RAP2.6L* (Asahina et al., 2011; Pitaksaringkarn, Ishiguro, Asahina, & Satoh, 2014a). As the cut heals, the asymmetric auxin response decreases (Asahina et al., 2011). Suppressing the function of either *RAP2.6L* or *ANAC071* inhibited wound healing (Asahina et al., 2011), suggesting that the differential auxin response across the cut is important for the reunion process. Wounding by laser ablation of cells in the root quiescent center also disrupts auxin flow and elevates auxin response markers one to two cell layers around the wound site within 3 h of cell death (Xu et al., 2006). Changes in cell fate followed this increase in auxin response (Xu et al., 2006), and these data are consistent with changes in auxin distribution and auxin response inducing a regeneration program.

Another possible mechanism to initiate graft healing involves detecting damage to cells. Cell wall damage or cell wall breaches are perceived by the plant and trigger a variety of defense and growth responses (reviewed by Nuhse, 2012). For instance, damage to the cell wall releases oligosaccharide fragments that are thought to be detected by sensors in the cell wall that regulate growth (Nuhse, 2012). Damage causing cell lysis can also change the mechanical properties of surrounding cells since plant cells contain considerable turgor pressure that is contained by the cell wall and the pressure exerted from neighboring cells (Schopfer, 2006). When cells are lysed in the shoot apical meristem, local turgor pressure is eliminated and microtubules reorient around a wound site to provide mechanical support (Hamant et al., 2008). Around the ablation site, cells also expand and the auxin efflux carrier PIN‐FORMED1 (PIN1) reorients (Hamant et al., 2008; Heisler et al., 2010). This reorientation could modify auxin transport to enhance a hormone response and contribute to wound healing. Ablation of the root endodermal cells triggers the neighboring pericycle cells to expand and divide but surprisingly in an auxin independent manner (Marhavy et al., 2016). Pericycle cell divisions give rise to lateral roots (Malamy & Benfey, 1997), yet ablation alone was insufficient to trigger lateral root formation. Only when ablation was combined with auxin was a lateral root formation program triggered (Marhavy et al., 2016), suggesting that the combination of hormonal and mechanical cues is important to trigger organ formation. Likewise, both mechanical and hormonal cues could be critical to initiate regeneration at the graft junction.

## 4 CELL ADHESION AND CELL DIVISION

During the regeneration process, tissues adhere, cells begin to divide and cell differentiation proceeds. Initially, thousands of genes are differentially expressed in *Arabidopsis* hypocotyls 1 day after grafting, including 306 genes that are thought to be grafting specific (Yin et al., 2012). In cut *Arabidopsis* hypocotyls, the transcription factor *WOUND INDUCED DEDIFFERENTIATION 1* (*WIND1*) activates around the wound within several hours of cutting. Its expression promotes cell dedifferentiation and cell division to form wound‐induced callus (Iwase et al., 2011) (Figs 2). In grafted *Arabidopsis* hypocotyls, *WIND1* is strongly upregulated above the graft junction, and then later below (Melnyk, Schuster, Leyser, & Meyerowitz, 2015). *WIND1* appears to act upstream of signaling of the plant hormone cytokinin, as cytokinin mutants block the effect of *WIND1* overexpression and the addition of cytokinin enhances the *WIND1* overexpression phenotype (Iwase et al., 2011). Cell division is also rapidly promoted during cutting and grafting. In cut inflorescence stems, markers associated with cell division are upregulated by 3 days as cortex and parenchyma cells divide (Asahina et al., 2011). Cell divisions are clearly important for healing since inhibiting cell divisions in cucumber by removing the cotyledons prevented wound healing (Asahina et al., 2002). In *Arabidopsis* hypocotyls, cell divisions occur within 2−3 days after grafting in the vascular tissues around the graft junction (Yin et al., 2012; Melnyk et al. 2015). Cell differentiation also occurs and cells rapidly lose and gain new cell identities. In cut root tips, cells lose expression of cell identity markers before regaining a similar or new identity (Xu et al., 2006; Efroni et al., 2016). Similarly, these cell identity markers changed location in healing tissues (Xu et al., 2006; Efroni et al., 2016), suggesting a dynamic dedifferentiation followed by differentiation process. At the graft junction, the endodermal‐specific marker *CASP1* activates 4 days after cutting and correlates with the reformation of the Casparian strip, consistent with restoration of normal vascular anatomy (Melnyk et al., 2015). Further work with cell identity markers will be informative to understand what cells are contributing to and important for the formation of the graft junction.

**Figure 3:**
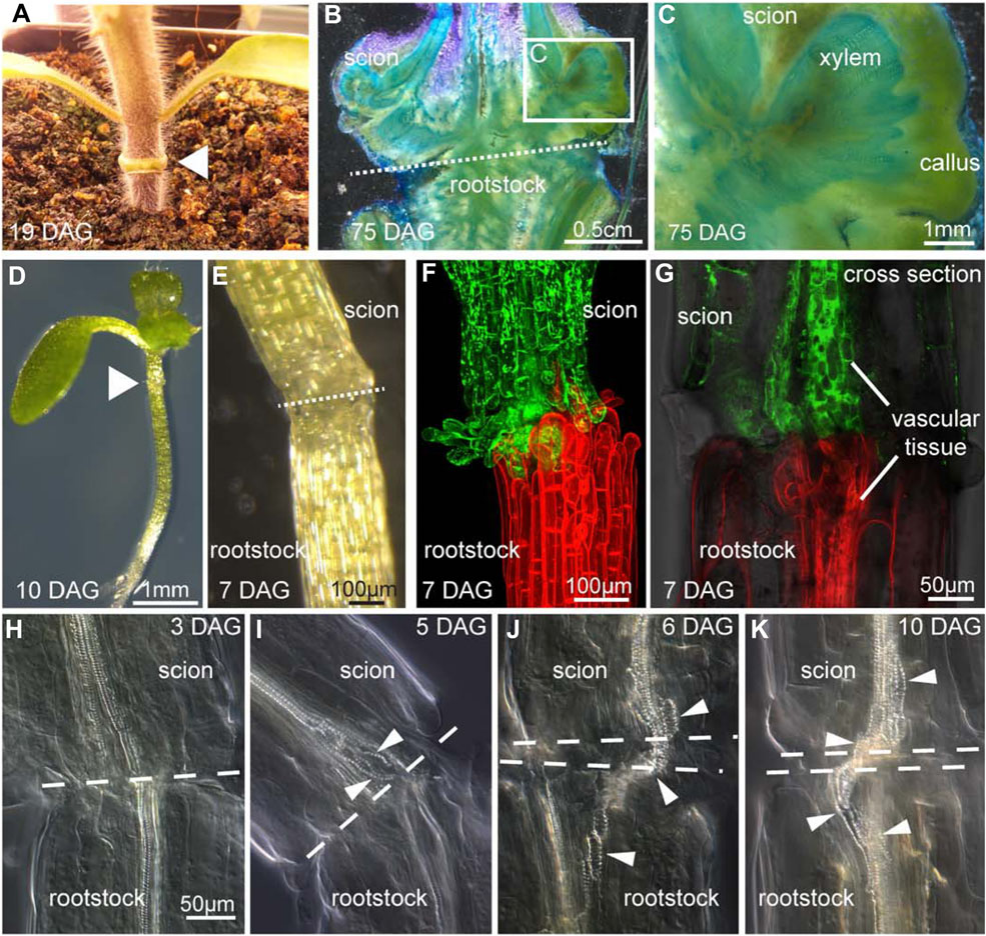
**Grafting in tomato and *Arabidopsis***. (A), (B), (C) Callus forms between the stock and scion of tomato grafts 19 or 75 days after grafting (DAG). Stems were cut and stained with toluidine blue which stains xylem light blue (B), (C) (images by Matt Jacobs). The triangle or dashed lines denote the graft junction. (D)−(G) Less callus forms at *Arabidopsis* grafts and instead, the vascular tissues expand and divide (G). (F), (G) Scions expressing green fluorescent protein are grafted to rootstocks expressing tomato fluorescent protein. The triangle or dashed lines denote the graft junction. (H), (I), (J), (K) New xylem elements (denoted by white triangles) form across the graft junction (dashed lines) between 5 and 6 DAG in *Arabidopsis thaliana*. Image reprinted from Melnyk et al. (2015) with permission from Elsevier.

At the graft or cut site, cell wall materials are deposited including pectins that are thought to cause opposing tissues to adhere and strengthen the graft junction (Jeffree & Yeoman, 1983; Asahina et al., 2002; Pina, Errea, & Martens, 2012). Cell‐modifying enzymes are induced in cut stems, including *XTH19* and *XTH20*, members of the xyloglucan endotransglucosylase/hydrolases family that construct or restructure cell walls (Pitaksaringkarn et al., 2014b). The early attachment process appears to be non‐specific, as cut stems will adhere to inert objects (Moore & Walker, 1981). Incompatible grafts also initially show attachment dynamics similar to compatible grafts, but after several days attachment weakens whereas compatible grafts continue to strengthen (Box 2) (Moore, 1983). Cells at the cut expand to fill the gap and resemble protoplasts with very thin cell walls (Jeffree & Yeoman, 1983; Melnyk et al., 2015). In most plant species, callus forms and fills the gaps between adhering tissues to allow contact between opposing tissues (Aloni et al., 2010; Garner & Bradley, 2013) (Figs 2 and 3). Surprisingly, grafted *Arabidopsis* hypocotyls produce little callus (Yin et al., 2012), although when cut and not grafted the cut top produces large amounts of wound‐induced callus (Melnyk et al., 2015). Reducing the ability to form wound‐induced callus in *Arabidopsis* by suppressing *WIND1* targets had no effect on phloem connection (Melnyk et al., 2015). Together, these data suggest that callus formation at the graft is not an absolute requirement for successful graft formation or vascular connection. Callus formation is important to promote grafting for many plants (Garner & Bradley, 2013), indicating that the importance of callus may be species specific. Whether callus is a cause or consequence of successful grafting remains unknown. The cell types that give rise to callus at the graft junction are also unknown, but cells in the vascular tissue are good candidates as *WIND1* is strongly expressed there (Iwase et al., 2011; Melnyk et al., 2015). Other layers may also be involved, since in *Arabidopsis* grafts the outer cell layers undergo large amounts of cell expansion (Melnyk et al., 2015) that may assist to fill gaps and promote tissue adhesion. Callus is typically formed during wounding or during infection by certain plant pathogens (Ikeuchi et al., 2013) but it can also be formed when plant tissues are placed on media containing high levels of auxin known as callus‐inducing media (CIM) (Ikeuchi et al., 2013). Callus formed on CIM takes a developmental program similar to lateral root development (Sugimoto, Jiao, & Meyerowitz, 2010) and, in the root, callus forms from the same cells that give rise to lateral roots, the xylem pole pericycle (XPP) cells (Malamy & Benfey, 1997; Atta et al., 2009). On the other hand, wound‐induced callus takes a different developmental fate that does not appear to be related to root formation (Iwase et al., 2011). It is likely that callus formed at the graft junction will be most similar to wound‐induced callus, but transcriptional analyses are required for confirmation. Intriguingly, the *ABERRANT LATERAL ROOT FORMATION 4* (*ALF4*) gene that blocks CIM‐induced callus formation (Sugimoto et al., 2010) is also important for graft formation (Melnyk & Meyerowitz, 2015), suggesting that at some level the CIM‐induced callus and grafting are similar. It may be that both require XPP cells, there is some transcriptional overlap between these processes, or both require auxin response as *ALF4* is important for auxin perception (Celenza, Grisafi, & Fink, 1995; DiDonato et al., 2004). When *Arabidopsis* root tips are excised, different cell types including the vasculature and endodermis contribute to reforming the stem cell niche and root tip (Efroni et al., 2016). Thus, XPP cells might be important for graft formation but it is likely that multiple cell types including the cambium contribute to healing.

Several days after grafting, the pectin layer deposited between the opposing tissues thins and plasmodesmata form between the adjoining cells of the graft (Jeffree & Yeoman, 1983; Kollmann & Glockmann, 1991). Continuous and connecting plasmodesmata are found where aligned vascular tissues connect, whereas in the cortex and in misaligned tissues, non‐connecting and discontinuous plasmodesmata form (Kollmann, Yang, & Glockmann, 1985). Plasmodesmata can form between grafts of unrelated species (Kollmann & Glockmann, 1985) and might be important for promoting graft formation (Jeffree & Yeoman, 1983). Recent reports revealed that genetic material including nuclear, mitochondrial, and chloroplast genomes combine from the rootstock and scion at the graft junction to generate cells with genomes from both graft parents (Stegemann & Bock, 2009; Stegemann, Keuthe, Greiner, & Bock, 2012; Thyssen, Svab, & Maliga, 2012; Fuentes, Stegemann, Golczyk, Karcher, & Bock, 2014; Gurdon, Svab, Feng, Kumar, & Maliga, 2016). Fuentes et al. (2014) suggest that nuclei migrate short distances between scion and stock via plasmodesmata because, although nuclei are from both parents, chloroplasts are only from one parent, suggesting an absence of cytoplasmic mixing. Another possible explanation for these phenomena is cell fusion since during fusions of plant cells lacking cell walls (called protoplasts) the cytoplasms often segregate non‐randomly (Evans, 1983). In cell fusions between two varieties of *Nicotiana*, chloroplasts are present only from one parent but mitochondria recombine with each other (Belliard & Pelletier, 1978) similar to what has been reported at the graft junction (Gurdon et al., 2016). Protoplast‐like protrusions form at the graft junction (Jeffree & Yeoman, 1983; Melnyk et al., 2015) and it is possible that cells from the graft parents fuse together at the graft junction. Ultimately, more work is needed to address this question and to discover the exact mechanism for genomes combining at the graft junction since this technique presents an important biotechnological innovation for creating hybrid plants asexually.

## 5 THE FORMATION OF VASCULAR TISSUE

The formation of vasculature connections between tissues is one of the hallmarks of grafting success. Although it does not guarantee success in the long term, a failure to form vascular connections will almost always lead to graft failure (Yeoman, Kilpatrick, Miedzybrodzka, & Gould, 1978; Aloni et al., 2010). In some exceptional instances, grafts between distantly related species such as *Arabidopsis* and tomato attach and grow despite no functional vascular connections (Flaishman et al., 2008). It is doubtful that these grafts would succeed in the long term since nutrient and hormone transfer between tissues would be inefficient. Despite the importance of vascular formation across the graft junction, we are only beginning to understand how this process occurs. Work in *Arabidopsis* has been extremely informative. Using functional assays that measure the movement of fluorescent molecules it was found that phloem connections form 3−4 days after grafting whereas xylem connections form 6−8 days after grafting (Melnyk et al., 2015). Movement through the xylem correlated closely with the appearance of continuous xylem elements across the graft junction, demonstrating that functional assays were a reliable and relatively easy way to monitor vascular connectivity (Melnyk et al., 2015) (Fig. 3). Auxin and cytokinin response increased at the graft junction during the healing process and this response was specific to the vascular tissues (Yin et al., 2012; Wang et al., 2014; Melnyk et al., 2015). The elevated auxin response appeared at a similar time to the phloem connection, whereas the elevated cytokinin response occurred at a similar time to the xylem connection (Melnyk et al., 2015). Whether cytokinin response is required for xylem connection is unknown at present, but evidence is emerging that auxin response is important for phloem connection.

A role for auxin in regeneration has long been known. Pivotal experiments in cut *Coleus* stems revealed that xylem formation around the wound could be reduced by the removal of upper leaves and restored by the application of auxin to the sites of the missing leaves (Jacobs, 1952). Analogous experiments with grafted pea plants revealed that xylem formation across the graft was blocked when the majority of the shoot was removed, but applying auxin in place of the missing shoot allowed xylem to form (Sachs, 1968). Auxin is also important for *in vitro* grafting assays. Here, two stem segments are attached in an agar dish and the formation of vascular connections and attachment strength are monitored (Fig. 2) (Parkinson & Yeoman, 1982). In the *in vitro* grafting system, the addition of apical auxin was critical to form new vasculature between the stem segments, whereas the addition of synthetic cytokinin enhanced vessel formation but only in the presence of auxin (Parkinson & Yeoman, 1982). Cytokinin also enhanced phloem regeneration in cut *Coleus* vascular bundles (Aloni, Baum, & Peterson, 1990). The addition of the hormone gibberelic acid (GA) inhibited graft formation (Parkinson & Yeoman, 1982), which contrasted with a requirement for GA for cell division during tissue reunion in cut tomatoes and cucumbers (Asahina et al., 2002). Thus, cytokinin appears to enhance vascular formation but the exact role for GA during vascular formation remains elusive. Sugar also plays a role since adding sugar to the grafting medium is important for grafting success when cotyledons are removed from grafted *Arabidopsis* (Marsch‐Martinez et al., 2013). Sugar and auxin also induce vascular tissue formation in isolated callus. Here, low concentrations of auxin induced phloem, whereas higher concentrations induced both xylem and phloem elements (Aloni, 1980). The addition of sugar is critical for inducing vasculature in callus (Wetmore & Rier, 1963) but notably, vascular bundles formed on callus are not continuous elements but scattered nodules or bundles (Wetmore & Rier, 1963; Aloni, 1980). Thus, simply adding auxin and sugar to an undifferentiated cell mass is not sufficient to induce continuous vascular elements, suggesting that although these substances are critical, other processes or hormones are also required.

To better understand the hormonal requirements for vascular formation, 45 *Arabidopsis* genotypes mutant in various hormone pathways including auxin, cytokinin, and ethylene were tested using fluorescent mobility grafting assays (Melnyk et al., 2015). Despite the strong developmental defects of some of these mutants, such as the dwarf and stunted *axr2‐1* mutant, very few mutations affected phloem connection indicating that young *Arabidopsis* hypocotyls graft robustly (Melnyk et al., 2015). Only four genotypes with mutations in the auxin signaling pathway substantially delayed phloem connection including mutations in *AUXIN RESISTANT 1* (*AXR1*), a *TRANSPORT INHIBITOR RESPONSE 1*/*AUXIN SIGNALING F‐BOX* (*TIR1*/*AFB*) triple mutant, *INDOLE‐3‐ACETIC ACID INDUCIBLE 18* (*IAA18*) and *ALF4* (Melnyk et al., 2015). Testing the spatial requirements for *AXR1* and *ALF4* revealed that they were required close to the graft junction and only in the tissue below the junction suggesting that the rootstock is more responsive to auxin allowing it to effectively perceive shoot‐derived auxin to promote reconnection. Mutating *AXR1* also on the shoot side of the graft surprisingly rescued phloem connection (Melnyk et al., 2015), possibly due to the elevated levels of auxin present in *AXR1* mutants (Nordstrom et al., 2004; Jones et al., 2010). Consistent with a role for auxin in tissue reunion, suppressing *AUXIN RESPONSE FACTOR 6* and *AUXIN RESPONSE FACTOR 8* activity inhibited cell division and healing of a cut (Pitaksaringkarn et al., 2014a). Taken together, these results indicate that auxin plays a critical role in vascular connection and that an asymmetry in auxin response occurs at the graft junction. Notably, few mutants affecting leaf vein development have been identified in *Arabidopsis* considering the complexity of this phenomenon (Sachs, 2000). Vein formation thus appears to be a robust process and it is likely that a similar robust pathway operates to form vasculature at the graft junction.

Further details regarding vascular development across the graft junction remain unknown, but work using cut stems where the vasculature has been severed has shed light on how this might occur. In cut *Zinnia* stems, expression of markers specific to xylem (*TED3*) and phloem (*ZeHB3*) activated within 48 hours of cutting, more strongly above the cut site than below (Nishitani, Demura, & Fukuda, 2002). Cambial activity is also induced more strongly above the cut, and the cambium as well as surrounding parenchyma cells differentiate into phloem and xylem precursor cells (Nishitani et al., 2002). Similarly in cut pea roots, the differentiation of vascular tissues occurs in the cambium and proceeds into the cortex and surrounding parenchyma (Hardham & McCully, 1982; Schulz, 1986). In *Zinnia* stems and pea roots, the differentiation of parenchyma cells to xylem did not require cell division, whereas the differentiation to phloem depended on one to three cell divisions (Schulz, 1986; Nishitani et al., 2002). Thus, in cut stems multiple cell types divide and differentiate to heal the wound. The role of auxin is also important for regeneration. Severing the vascular strands in pea stems induced broad expression of the auxin efflux protein PIN1 that narrowed and oriented around the wound with time, after which xylem cells differentiated (Sauer et al., 2006). Similar results were observed in cut *Arabidopsis* stems (Mazur, Benkova, & Friml, 2016) consistent with a role for auxin transport promoting vasculature formation around and across the wound. Clearly, tissue regeneration is complex since, in *Arabidopsis*, hundreds of genes are upregulated 1 day after cutting including genes involved in classic stress response pathways such as those mediated by jasmonic acid and ethylene (Asahina et al., 2011; Pitaksaringkarn et al., 2014a). It will be informative to compare such datasets to grafting datasets (Yin et al., 2012) to discover the similarities and differences between these processes. A better understanding of the wound response in plants will be pivotal for understanding how plants graft, as it is likely that self‐grafting and healing severed vascular strands proceed via the same regeneration pathway.

## 6 VEIN FORMATION: LESSONS FROM OTHER TISSUES

Although our understanding of vascular formation at the graft junction is only beginning to develop, other related processes could prove highly informative for understanding how plants graft. Vein connection across a cut or graft is not a unique example of vein connection in plants. Veins connect within a plant during normal development when leaves, flowers, roots, and other new organs form and connect to the existing vasculature. Leaves present an elegant model as the pattern of vein formation is easily observed. In *Arabidopsis* leaves, the majority of veins are connected to each other and form a closed vascular network (Scarpella, Francis, & Berleth, 2004). Veins are formed in leaves from homogeneous and undifferentiated subepidermal tissues that differentiate to vascular tissue (Scarpella et al., 2004). Auxin is produced at the leaf tips (Aloni, Schwalm, Langhans, & Ullrich, 2003) and concentrates into channels by the action of auxin transporters, including the efflux protein PIN1, before draining out of the leaf (Scarpella, Marcos, Friml, & Berleth, 2006). Auxin response increases in these channels and activates *MONOPTEROS*, which in turn activates *AtHB8* expression (Wenzel, Schuetz, Yu, & Mattsson, 2007; Donner, Sherr, & Scarpella, 2009). *AtHB8* is a marker for procambial activity, and it is thought that once *AtHB8* expression is activated cell fate is fixed to become vascular tissue. New veins emerge from existing veins before connecting to other veins (Scarpella et al., 2004) whereas xylem differentiation proceeds from the base of the leaf to the apex (Kang & Dengler, 2004). These observations indicate that new vascular tissues typically emerge from existing vascular tissues. The same could also be true for grafted tissues since alignment of the cambial tissues that give rise to veins is important for grafting success (Garner & Bradley, 2013). Notably, to date the majority of genes expressed during leaf vein formation have also been expressed during root vascular development (Gardiner, Sherr, & Scarpella, 2010; Gardiner, Donner, & Scarpella, 2011). In addition, root vascular formation expression profiles have been useful to identify genes involved in leaf vein formation (Gardiner et al., 2011). Thus, substantial similarities exist between leaf and root vein development, and it is possible that veins formed across the graft junction use similar or identical genetic pathways. The alignment of cambial tissues is critical for grafting success (Garner & Bradley, 2013), and during stem and root thickening in plants the cambial tissue divides and differentiates to xylem or phloem consistent with the idea that cambium gives rise to mature vascular tissue during both grafting and normal growth.

Vein formation also occurs during embryogenesis, during growth at the shoots and roots (primary growth), and during stem and root thickening (secondary growth). Many of these vein formation processes are well described. For instance, various hormones and transcription factors are important for provascular formation in the embryo and cambium/procambium formation in leaves, roots, and stems during primary growth (reviewed by Miyashima, Sebastian, Lee, & Helariutta, 2013; Furuta, Hellmann, & Helariutta, 2014). Likewise, cambium and procambium differentiation to xylem and phloem involves various well described hormone and transcription factor mediated pathways (reviewed by Heo, Roszak, Furuta, & Helariutta, 2014; Ruzicka, Ursache, Hejatko, & Helariutta, 2015). In many instances, similar pathways or genes operate in these various tissues. A future aim will be to determine whether similar or identical pathways occur at the graft junction and how they might be involved with forming the vasculature across the graft junction.

## 7 COMPATIBILITY AND INCOMPATIBILITY

Much of our understanding of grafting and wound healing is from studying tissue reunion from the same genotype. Grafting plants with identical genetic backgrounds is not commonly practised in horticulture. Instead, plants are grafted to different cultivars or species. In many instances, grafts between different species have varying success rates; in general, the more distantly related the species, the lower the odds of grafting success. Grafting within a genus is often successful, but within a family success is unusual. Exceptional instances include the Solanaceae (tomatoes, potatoes, and tobacco) and cacti families, which often graft successfully within their respective families (Yeoman et al., 1978; Lewis & Alexander, 2008). Notable examples are tomato scions grafted to potato rootstocks (the commercially available TomTato® or Ketchup ‘n’ Fries™ plant), eggplant scions to potato rootstocks (Egg & Chips® plant), or ornamental red ball cacti scions grafted to green cactus stocks (Fig. 1). Some plant species are incapable of grafting to themselves, such as monocots, whereas other plants cannot be grafted to each other but can be grafted to a common third plant, giving rise to a grafting process called double‐working. Here an intermediate compatible tissue is grafted between two genotypes to allow the graft to take (Garner & Bradley, 2013). The reason for much of this compatibility and incompatibility remains unknown but themes are beginning to emerge.

Here, I discuss two types of incompatibility: short and long term. In short‐term incompatibility vascular strands fail to form across the graft junction and the graft is short lived, surviving less than a couple of weeks or months. One example is *Arabidopsis* grafted to tomato. Although some grafts will take, no functional vascular connections are formed (Flaishman et al., 2008) and it is unlikely that this type of graft would survive in the long term. In horticulture, quince is often used as a rootstock for pear scions, and compatible cultivars show phloem connectivity within 10−20 days after grafting (Espen, Cocucci, & Sacchi, 2005). Certain incompatible combinations show limited phloem connectivity even 30 days after grafting and xylem differentiation is delayed (Espen et al., 2005). This type of incompatibility is presumably quite common between unrelated species and is straightforward to identify due to poor growth or death. A second type of incompatibility is long term. Here, grafts adhere and vascular strands form across the graft junction. The grafted scion initially grows well, and it can take months or years before problems emerge, such as the graft junction breaking or the scion no longer growing vigorously (Garner & Bradley, 2013). Even here there can be early signs of incompatibility. In grafts between plums and apricots, both compatible and incompatible varieties adhere, produce callus and form functional vascular connections, but incompatible grafts have slower callus differentiation rates to cambium (Errea, Felipe, & Herrero, 1994; Pina et al., 2012). Incompatible grafts typically have higher levels of stress responsive compounds including reactive oxygen species and in some instances lower transcript levels of anti‐oxidant genes (Aloni et al., 2008; Irisarri, Binczycki, Errea, Martens, & Pina, 2015). Phenolic compounds and the enzymes responsible for their biosynthesis are also higher in incompatible grafts (Pina & Errea, 2008; Pina et al., 2012). Clearly, grafting is a stressful process and whether the increased stress response is a cause or consequence of graft failure remains to be determined. One instance of graft failure where mechanistic insight exists is between some incompatible pear and quince grafts. The quince rootstock produces a compound, prunasin, that moves into the pear scion where enzymes break it down to release cyanide. The cyanide causes tissue necrosis and the graft fails (Gur, Samish, & Lifshitz, 1968). Some pear varieties are compatible with quince though and here placing a short segment of compatible stem between the two incompatible plants (double‐working) allows the graft to succeed (Garner & Bradley, 2013).

Much work still needs to be done to determine the basis for compatibility. It is likely that no single mechanism will be responsible for determining incompatibility, but instead multiple mechanisms work together to allow a graft to succeed. One theory by Randy Moore states that morphogens at the graft junction promote graft formation, such as auxin, whereas toxins inhibit graft formation, such as cyanide (Moore, 1984). There is a balance between the two and incompatibility occurs when the morphogens are overridden by toxins (Moore, 1984). Moore hypothesized that these compounds work at the systemic level since both auxin and prunasin are mobile compounds. A second theory by Michael Yeoman and colleagues (Jeffree & Yeoman, 1983; Yeoman et al., 1978) proposed that compatibility required cell‐to‐cell recognition of the opposing graft partner. Here, tissue adhesion and the formation of plasmodesmatal connection, such as during phloem connection, were needed for grafting to succeed. It is likely that both theories are important and both long‐range and local factors contribute to graft formation. For instance, blocking auxin response immediately below the graft junction is sufficient to delay grafting (Melnyk et al., 2015), indicating that a local and tissue‐specific recognition system exists in the rootstock to perceive a systemically produced signal from the scion (auxin).

One solution for understanding compatibility and incompatibility is to study plants that form vascular connections between completely unrelated species: the parasitic plants. Parasitic plants represent approximately 1% of flowering plant species and attach to and connect their vasculature to the host's vasculature to withdraw water and nutrients (Musselman, 1980; Westwood, Yoder, Timko, & dePamphilis, 2010). Parasitic plants form direct xylem connections to their hosts and some also form connections between their phloems (Musselman, 1980; Haupt, Oparka, Sauer, & Neumann, 2001). Interestingly, many parasitic plants infect completely unrelated plants, such as *Striga* (a dicot) infecting rice (a monocot) (Musselman, 1980). Other parasitic plants, such as *Phtheirospermum japonicum*, can infect a wide range of species including *Arabidopsis* and rice plants (Cui et al., 2016). It is not well understood how parasitic plants overcome these recognition barriers to infect their hosts. In some instances, host plants block parasitic plant infection via receptors that detect the parasitic plant and cause local cell death to avoid vascular invasion (Li & Timko, 2009; Hegenauer et al., 2016). It is possible that similar defense mechanisms are activated and contribute to cell death when unrelated plants are grafted. It is also unknown whether parasitic plants use a similar vascular formation mechanism as that present in plant grafting, but the processes are conceptually similar (Melnyk, 2016), and the potential for modifying a parasitic plant pathway to improve plant grafting should be investigated. Ultimately, work with grafted plants and parasitic plants could shed light on the process of self/non‐self recognition which we currently know little about.

## 8 CONCLUDING REMARKS

**Figure 4:**
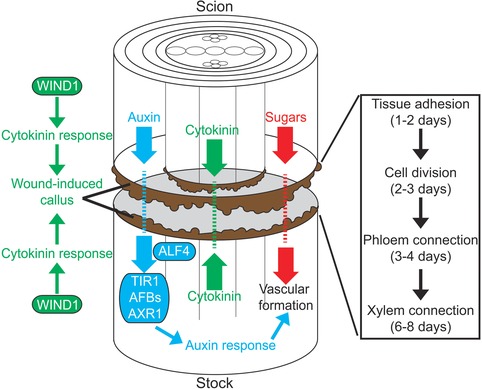
**A framework for graft formation in *Arabidopsis thaliana***. Upon cutting, auxin, cytokinin, and sugar transport are blocked at the graft junction and a wound healing pathway is activated. The *WIND1* pathway activates and produces callus whereas auxin response proteins (TIR1, AFBs, AXR1, ALF4) below the graft junction perceive scion‐derived auxin to promote vascular connection (Iwase et al., 2011; Melnyk et al., 2015). Vascular tissues divide and differentiate to reconnect the vascular tissues by 8 days after grafting (Melnyk et al., 2015). Wound‐induced callus is found throughout the cut surface. Time‐course data taken from Yin et al. (2012) and Melnyk et al. (2015).

Grafting is a fascinating process that we are only just beginning to understand. The use of grafting is becoming increasingly common since it easily improves vigor, disease resistance, and stress resistance without resorting to genetic modifications or lengthy plant breeding programs. The majority of grafting research studied commonly grafted species such as peaches, plums, grapes, and tomatoes (Jeffree & Yeoman, 1983; Errea et al., 1994; Espen et al., 2005; Cookson et al., 2013) but this research is hindered by a lack of genetic resources and the long generation times in these species. The ability to graft the model plant *Arabidopsis thaliana* to itself and its closely related species (Flaishman et al., 2008; Melnyk et al., 2015) should bring rapid advances in our understanding of grafting (Figs 3 and 4). One major question is whether the mechanisms that occur during *Arabidopsis* grafting will be the same as those found in commercial species and, if so, whether this information can be effectively transferred from *Arabidopsis* to these. It seems unlikely that different plant species have developed different mechanisms to graft but further work is needed to resolve this question.

The evolution of grafting remains mysterious. In some species, a type of natural grafting exists whereby physical contact and pressure cause stems to fuse, such as in English ivy and *Ficus virens* (Fig. 1). Stem fusion is unusual, although there is some indication that roots may more frequently fuse within and between plant species (Garner & Bradley, 2013). Grafting may have evolved from such an ability to fuse tissues, but more likely, grafting probably originated from a combined wound healing and vein formation mechanism. Most plants can effectively heal cuts and wounds by producing callus and differentiating new cells. Likewise, the accumulation of auxin induces vein formation in leaves and across cuts. Thus the flow of auxin and other hormones across the graft junction could induce vasculature formation in the callus to connect the graft. This idea does not explain differences in graft compatibility so understanding and discovering the fundamental differences in grafting success between species should be priorities. Likely candidates could include inefficient wound healing responses, differences in auxin responses, or the presence of toxins or substances in one plant that inhibit vascular formation in the other plant. One interesting example is the inability of monocots to graft. Although dicots and the more ancestrally related gymnosperms can graft, the majority of monocots do not graft efficiently (Muzik & La Rue, 1952; Garner & Bradley, 2013). The belief is that the absence of vascular cambium and/or scattered vascular bundles prevents grafting (Garner & Bradley, 2013). Some success is possible grafting with intercalary meristems (Muzik & La Rue, 1952) suggesting that tissue specificity and cell type could play important roles in the future to improve and broaden grafting.

The future for grafting looks bright as its use and applications continue to grow. With rootstock breeding efforts and automation techniques, billions of plants globally are now grafted (Lee et al., 2010). Increased efforts are needed to understand how plants graft and ultimately to understand the basis for compatibility and incompatibility. The ability of parasitic plants to fuse tissues and form vascular connections to unrelated species could prove highly informative to understanding how to overcome incompatibility barriers. Likewise, a better understanding of how parasitic plants overcome these barriers could be used to combat parasitic plant infections. Through work using *Arabidopsis* and translating this information to commercially important plants, major breakthroughs are possible. Furthermore, such advances will provide mechanistic insight into processes such as regeneration, cell wall biogenesis, hormone response, vascular development, biomechanics, and self/non‐self recognition that could greatly improve our understanding of fundamental plant biology.
